# Palladium‐induced granulomas analysed with inductively coupled plasma mass spectrometry

**DOI:** 10.1111/cod.12979

**Published:** 2018-03-01

**Authors:** Nick Marsidi, Jos H. Beijnen, Esther J. van Zuuren

**Affiliations:** ^1^ Department of Dermatology Leiden University Medical Centre Leiden The Netherlands; ^2^ Department of Clinical Pharmacology The Netherlands Cancer Institute Amsterdam The Netherlands

**Keywords:** allergic contact, case report, foreign body, granuloma, ICP‐MS, palladium

## CASE REPORT

**Figure 1:**
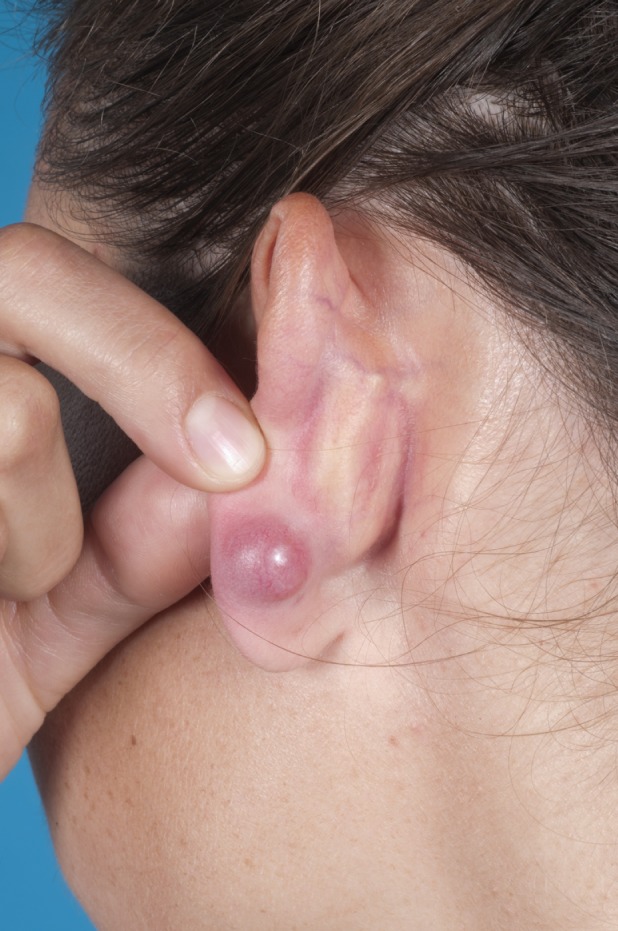
Symmetrical nodes on both earlobes

A 28‐year‐old female was referred to our dermatology clinic because of persistent swelling of the earlobes several months after ear piercing. Clinical examination showed symmetrical nodes on both earlobes (Figure 1). Histology showed epithelioid granulomas with a lymphocytic infiltrate, as seen in sarcoidosis and foreign body reactions. There were no further signs of sarcoidosis (normal chest X‐ray and normal angiotensin‐converting enzyme findings) or foreign material. Patch testing with the European baseline series and a dental series (including various metals) was performed. Positive reactions to nickel sulfate 5% pet. [+ on day (D) 2 and D3] and palladium chloride 1% pet. (+ on D3) were observed. Four weeks after the patch test, a persistent reaction on the patient's back remained at the palladium test site. A biopsy showed epitheloid granulomas similar to those previously seen in the excised nodes.

Inductively coupled plasma mass spectrometry (ICP‐MS) (ICP‐quadrupole‐MS, Varian 810‐MS) detected palladium (^105^Pd) in all of our skin samples (thickness, 4 μm) and showed a 3‐fold higher signal than that from skin samples of control patients. The content of ^105^Pd in each sample was semiquantitatively determined to be 0.6 ng of ^105^Pd per sample.

## DISCUSSION

Similar cases have been described in the literature, in which granulomas have been found in the earlobe with a positive reaction to palladium after patch testing.^1^, ^2^, ^3^, ^4^ The presence of palladium has been confirmed in earrings, and is a likely source of this reaction.^4^ The authors described the combination of these findings as an allergic contact granuloma, but the skin biopsies have never been tested for the presence of metals.

This raises the question of whether contact sensitization alone is enough to cause granulomas, as the clinical relevance of palladium hypersensitivity remains uncertain. Tillman et al. investigated the wearing of palladium‐coated earrings, and found no skin reactions in 40 subjects who showed positive patch tests to palladium.^5^ Another possibility is that palladium permeates through the skin, causing a foreign body reaction. Skin penetration and permeation of palladium nanoparticles in the epidermis and dermis has been shown in damaged skin.^6^ Other cutaneous granulomas have also been reported to be induced by beryllium and zirconium, and in these cases metals have been found in the skin.^3^, ^4^

To the best of our knowledge, we present the first case in which epithelioid granulomas have been found in the earlobes with a positive patch test reaction to palladium and the confirmed presence of palladium in the skin samples. Although the pathogenesis remains unclear, our findings suggest that palladium might induce granulomas via a foreign body reaction in patients with positive palladium contact allergy.

### Acknowledgements

We thank Dr A.P.M Lavrijsen for her support.

### Conflict of interest

The authors declare no potential conflict of interests.
